# The German System. How the New Insurance Regulations Affect the German Hospitals

**Published:** 1912-02-10

**Authors:** 


### HOSPITALS AND STATE INSURANCE.

### The German System.

### HOW THE NEW INSURANCE REGULATIONS AFFECT THE GERMAN HOSPITALS.

By OUR SPECIAL COMMISSIONER.

In foregoing articles (The Hospital, June 17, 24, July 1, and 8, 1911) we outlined the scope and nature of the various laws regarding Invalidity, Sickness, and Accident Insurance with reference to their special effect upon the hospital system in Germany. It now remains to outline, briefly, the modifications which have been recently introduced in these various Acts and to discuss in what way they have a bearing on hospital politics. The text of the new modifications is comprised in a volume lately issued by the authorities in Berlin.* This volume contains no fewer than 1,805 paragraphs, and replaces the various Novellen which have been issued from time to time as memoranda regarding the scope and application of the law. It consists of five separate sections, of which the first deals with the administration of the funds, the election of office-bearers and committee men, and the regulation of the powers of local authorities under the Act. The second, third, and fourth books deal exhaustively with Sickness, Accident, and Invalidity and "Hinterbliebenen" insurance respectively; the fifth section discusses the relationship between these various classes of insured persons; while a sixth book deals with the methods to be followed in arbitration and judicial processes arising out of the interpretation and application of the Acts.†

With regard to the composition of local committees, which are in some ways analogous to those which are provided for under the Health Committees in Mr. Lloyd George's Act, the most striking modification is the acceptance by the Legislature of the suggestion that women should be represented on the boards. Under the new regulations women are eligible for membership of such committees or boards; they must be appointed by the local associations or societies in precisely the same manner as the male members are now appointed—namely, by ballot. Members are paid working expenses and compensation for loss of wages incurred by service on the board. This is a step in the right direction, but is still only a step. A member of a committee which has charge of the local adjustment of the fund must necessarily devote a great portion of his or her time to the service of the fund, and it is only fair that reasonable compensation, and not merely compensation for loss of wages, should be paid to such a member. Certain minor modifications are made in the public insurance authorities. Thus the official Versicherungsamter are responsible for the distribution of the funds, and for making official reports to the central bureau. The chairman of the local authorities board is also the chairman of the Versicherungsamt, and may appoint substitutes who act for him in his absence. Such substitutes are chosen from among "those who have shown that they have an active interest in, and knowledge of, the insurance system generally"—a pretty wide definition. Only men are permitted to act as chairmen or substitute officers. The Imperial Insurance Department (Reichsversicherungsamt) is placed at Berlin, and is made into an Imperial Department responsible to the Chancellor and the Bundesrat. The Emperor appoints the president and the higher office-bearers, who hold office for life, on the recommendation of the Bundesrat; the other officials are appointed by the Imperial Chancellor. In addition there are thirty-two other members who are not permanent officials. Of these eight are chosen by the Bundesrat and twelve by the employers, while the remaining twelve are representatives of the insured employés. The appointments are made by ballot, and only males are eligible as office-bearers. The regulations with regard to the modified local or Landesversicherungsamt are of little general interest.

When we come to the modifications of the Invalidity and Sickness Acts we are at once struck by the fact that the Legislature has taken pains to obviate the difficulties which the loose phrasing of the older Acts brought about, and to embody many of the suggestions which have been made from time to time to amend the Acts. Formerly, for example, the law stated that certain persons employed in certain trades were liable to pay insurance premiums, and there existed considerable doubt as to what classes of persons were liable. This led, as we have already shown in a previous article, to extensive litigation, with the result that the interpretation of the Acts became a matter of considerable difficulty, in view of the various legal decisions which had emanated from the courts. The new regulations define the classes of persons liable to pay insurance premiums very distinctly. Seven categories are enumerated. In the first are labourers, labourers' helps, apprentices, pupils, servants (domestic servants); in the second, those employed in various trades, overseers, and others who are similarly employed, provided their employment constitutes their chief or main source of income. In the third section are junior employés of similar trades, apprentices, or pharmaceutical assistants; in the fourth, employés of theatres or orchestras, no matter whether or not their work is of an artistic character (ohne Rücksicht auf den Kunstwert der Leistungen); in the fifth teachers and instructors; in the sixth those employed in household duties; and in the seventh category the crews of German vessels. The income limit is raised from two thousand marks annually to two thousand five hundred marks—a figure which, for economic reasons which it is unnecessary to detail here, corresponds closely with Mr. Lloyd George's limit of £160.

Those who are employed by the State, or by the Municipalities, or by the insurance societies themselves, provided they are guaranteed sick pay by reason of such employment (as is invariably the case) are excluded. This is an interesting modification, which we submit to the Chancellor of the Exchequer as worth incorporating in the English Act, so as to meet the claims of hospital authorities who already guarantee such sick pay, or its equivalent, accepted by the German modifications, adequate treatment while sick, to nurses or employés. Similarly teachers or lecturers in public schools who have the same guarantee are excluded. Private corporations and firms who make similar provision for their employés may apply to the central authority for exemption, which may be granted to their employés if sufficient proof has been shown that such guarantee is effective, and that the interests of the employés are safeguarded by a sick fund or by proper provision for their treatment and assistance when they are incapacitated. Power is given to the central authority to widen the class of these exemptions

* Reichsversicherungsordnung nebst Einführungsgesetz. Textausgabe mit Einleitung und Sachregister (Berlin, 1911).

† "Die Reichsversicherungsordnung"; *Zeitschrift für Krankenanstalten.* No. 33, 1911.

on analogous grounds—an interesting provision, which is certainly one of the most important in the modifications since it opens a wide field for co-operation between working men and employers, while the interests of both sections are carefully safeguarded by the central authority.

Free of insurance duty are soldiers who, although they may in private life be liable to pay insurance premiums by reason of the fact that they are employés falling under one or other of the above-mentioned categories, are serving with the colours; persons who are in the service of the State or local authorities under the provisos already enumerated; persons who are occupied in preparing themselves for future professional work which is to be their main source of income—nurses, attendants in the wards, house officers, and similar persons would be included under this exemption—and members of religious societies (deaconesses, school nurses, etc.). These specific exemptions go far to meet the wishes of the hospital authorities, who have hitherto had to pay for the insurance of their employés under the old Acts; they certainly obviate the risk of costly and circumlocutory legal processes to find out whether or not a hospital employé is bound to insure himself as an ordinary labourer, or whether he is exempt by reason of the fact that he exercises professional services and has a semi-scientific status.

### Definition of Sick Benefits.

The sick benefits under the new regulations are defined with praiseworthy and exemplary distinctness. The insured, for instance, is guaranteed Krankenpflege, which, however, means more than mere nursing, as the name implies. It is specifically stated that Krankenpflege includes medical treatment, together with medicine and nursing and all needful adjuncts, such as spectacles, surgical apparatus, trusses, and minor necessities. The insured person is further guaranteed sick pay. Such benefits are continued for a period not exceeding twenty-six weeks from the commencement of the term of sickness. Express powers are given to the Krankenkass to guarantee hospital care and treatment in lieu of ordinary sick benefit in cases where such care seems called for. When more than one hospital is available the invalid may be allowed to choose his own institution, provided the hospital agrees to admit him. A special clause (371), however, gives the society power to limit the choice of hospital in certain cases—*i.e.*, in cases of infectious disease or of consumption. Where admission into a hospital is, for various reasons, impossible, the society may extend private nursing treatment to the insured and may enlist the services of a duly-qualified nurse.

The societies may, under special conditions, extend the term during which sick benefit is allowed to a period not exceeding one year. They may further extend to the insured the privileges of convalescent treatment in a special institution, in order to prevent permanent disablement, which may be averted by suitable orthopædic or other treatment. An important proviso is that such further help may be disallowed when there are grounds for believing that the insured has wilfully contributed, by neglect or otherwise, towards the inception of the condition that threatens disablement.

### The Position of Hospital Employés.

Obviously, the most important fact is that under the new regulations the position of hospital employés, in so far as their contributions to the funds are concerned, is definitely stated. The definitions contained in the exemption clauses make it clear that hospital nurses, male or female, and sick-room attendants, together with employés in the special departments, are no longer liable to pay insurance premiums. There still remains a comparatively large class of hospital employés whose position is somewhat vague. These are the gardeners, the laundry employés, the electricians, and the casual hospital labourers, laboratory attendants, out-patient officers, the man who winds the clocks—in institutions which do not possess the felicity of an electrically-controlled system—and the man who directs visitors at the entrance lodge. In so far as these are labourers under the Act they are liable to pay insurance premiums. But it is a well-established fact that such individuals have a prior claim on hospital treatment; the nature of their work, performed under the eyes of the hospital authorities, makes it possible for them to be treated early and effectively in cases of illness. It is true such treatment is not in the majority of cases definitely guaranteed, as is done in the case of nurses, members of the permanent staff, and residents. But none the less they are in a different position from that of outsiders. The probable way out of the difficulty would be for the hospitals to limit their staff of casuals, and to employ only recognised workmen, who may be looked upon as permanent employés of the institution, to whom the guarantees now extended to the nurses and other permanent employés will also be given. In that way it will be possible to obtain the exemption which the central authority is empowered to grant to public or private corporations who are in a position to provide adequate substitutes for the benefits given under the Act. The main point is that the possibility of such exemption has been granted. All that is required now is for the hospital authorities to combine and devise some means whereby advantage may be taken of the exemption clauses contained in the new modifications. To us in England the position is interesting. Like the German hospitals, our institutions practically offer a guarantee to their permanent employés, and it is only fair that the Legislature should take this fact into consideration and make allowance for it in apportioning the "liability" of hospital workers.

### A Necessary Exemption Clause.

We have already pointed out that under our Insurance Act, an added expense will devolve on our large institutions, who will have to pay big sums in insurance premiums while at the same time they will still remain, at least morally, responsible for the adequate treatment in hospital of their sick employés. This anomaly has been effectively met by the German modifications, and it is to be hoped that Mr. Lloyd George will yet see his way clear to incorporate a similar exemption clause in his Act when it comes up for discussion again. The raising of the income limit indirectly affects hospitals, since it will tend to increase the membership of the societies, and therefore to increase the number of their patients sent to hospital. The result of this will be an increase in the revenue derived from patients' payments, and to that extent the hospitals will find the increase of income limit beneficial. A section of the medical profession has protested against the raising of the income limit, just as in this country there has been a protest against the £160 limit, but public opinion in Germany is so distinctly in its favour that there is little likelihood that the clause will be rescinded. It is an absurdity that a person in possession of £125 a year should be excluded from medical benefits which are accorded to one who is in possession of £100, and there is already a strong feeling that even the former limit is not adequate. It must always be remembered, however, that the profession in Germany is in a much better position to benefit under the Acts, since hospital medical staffs are paid and are not purely honorary, as in England.

